# Redescription of *Pristidia cervicornuta* (Araneae, Clubionidae), with a first description of the female

**DOI:** 10.3897/zookeys.914.46909

**Published:** 2020-02-20

**Authors:** Jianshuang Zhang, Hao Yu, Yang Zhong

**Affiliations:** 1 School of Life Sciences, Guizhou Normal University, Guiyang, Guizhou, China Guizhou Normal University Guiyang China; 2 School of Biological Sciences, Integrated Mountain Research Institute, Guizhou Education University, Guiyang, Guizhou, China Guizhou Education University Guiyang China; 3 School of Nuclear Technology and Chemistry & Biology, Hubei University of Science and Technology, Xianning, Hubei, China Hubei University Wuhan China; 4 Hubei Collaborative Innovation Center for Green Transformation of Bio-Resources, Centre for Behavioral Ecology and Evolution, College of Life Sciences, Hubei University, Wuhan, Hubei, China Hubei University of Science and Technology Xianning China

**Keywords:** Diaoluo Mountains, DNA barcoding, morphology, sac spiders, taxonomy

## Abstract

*Pristidia cervicornuta* Yu, Zhang & Chen, 2017 is redescribed based on new material from the type locality, Diaoluo Mountains of Hainan Island, China. The female is described and illustrated for the first time. In addition, this paper further illustrates the male, and provides a supplementary description.

## Introduction

*Pristidia* Deeleman-Reinhold, 2001 is a relatively small genus, distributed exclusively in South East Asia, with only six species described so far, two of which are known from China (Yu et al. 2017; World Spider Catalog 2019). All *Pristidia* species were known from both sexes, except for *P. cervicornuta* Yu, Zhang & Chen, 2017.

**Figure 1.**
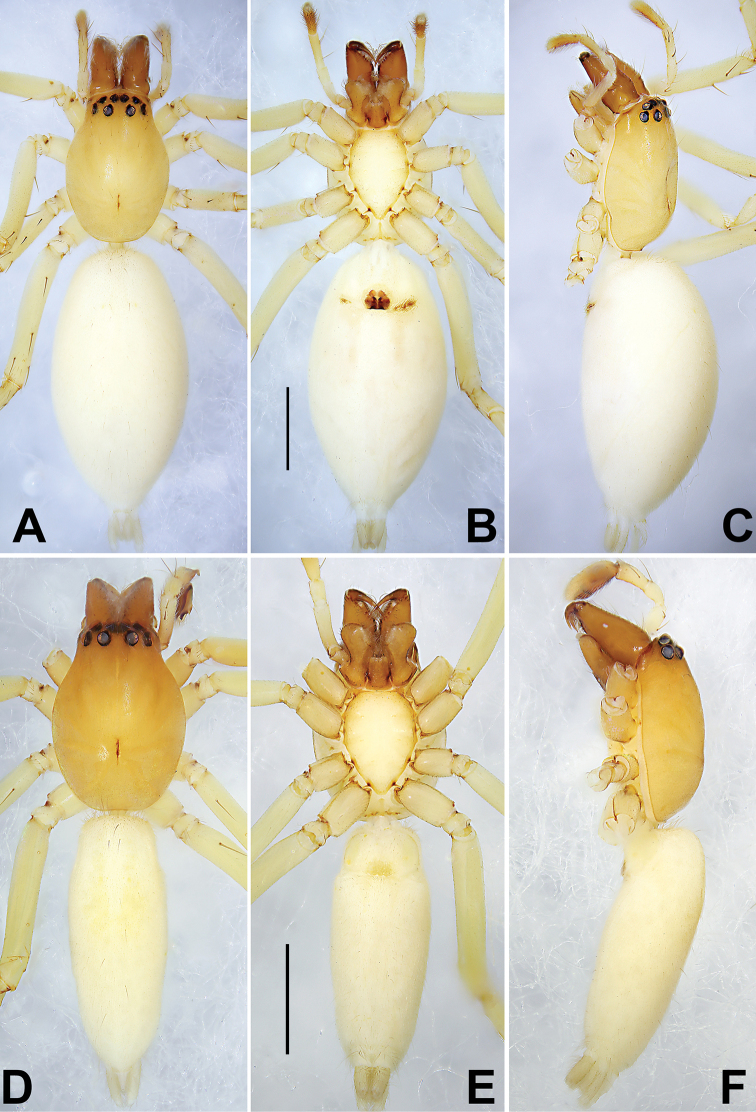
Habitus of *Pristidia cervicornuta* female (MGEU-PRI-18-001, **A–C**) and male (MGEU-PRI-18-018, **D–F**). **A, D** Habitus, dorsal view **B, E** ventral view **C, F** Lateral view; Scale bars: 1 mm (equal for **A–C**, equal for **D–F**).

*Pristidia cervicornuta* was first described based on two male specimens from Mt. Diaoluo of Hainan Island, China (Yu et al. 2017). Recently new material has been collected from the type locality containing both sexes. The males were identified as *P. cervicornuta* based on comparison with the type specimens. On the basis of the morphological characters (Fig. 1) and DNA barcoding (Table 1), we credibly matched the females and males together as *P. cervicornuta*. Additionally, we found some characters overlooked in the original description of the male. The aim of the current paper is to redescribe the male and report the female for the first time, providing detailed morphological descriptions and illustrations.

**Table 1.** Voucher specimen information.

**Voucher code**	**Sex**	**GenBank accession number**	**Sequence length**
MGEU-PRI-18-031 (YHCLU0006)	♂	MN897086	650bp
MGEU-PRI-18-032 (YHCLU0007)	♂	MN897087	650bp
MGEU-PRI-18-017 (YHCLU0008)	♀	MN897088	650bp

## Materials and methods

Specimens were examined with an Olympus SZX7 stereomicroscope; details were studied with an Olympus BX41 compound microscope. Female epigynes and male palps were examined and illustrated after being dissected. Epigynes were removed and cleared in warm lactic acid before illustration. Vulva was also imaged after being embedded in Arabic gum. Photos were made with a Cannon EOS70D digital camera mounted on an Olympus CX41 compound microscope. The digital images were taken and assembled using Helicon Focus 6.80 software package.

All measurements were obtained using an Olympus SZX7 stereomicroscope and given in millimetres. Eye diameters are taken at widest point. The total body length does not include chelicerae or spinnerets length. Leg lengths are given as total length (femur, patella, tibia, metatarsus, tarsus). The terminology used in text and figure legends follows Yu et al. (2017) and Yu et al. (2012).

A DNA barcode was also obtained for matching. A partial fragment of the mitochondrial cytochrome oxidase subunit I (CO1) gene was amplified and sequenced for three specimens, using the primers LCO1490 (5’-GGTCAACAAATCATCATAAAGATATTGG-3’) and C1-N-2776 (5’-GGATAATCA-GAATANCGNCGAGG-3’). For additional information on extraction, amplification and sequencing procedures, see Malumbres-Olarte and Vink (2012). All sequences were analysed using BLAST and are deposited in GenBank. The accession numbers are provided in Table 1.

All specimens (including molecular vouchers) are deposited in the Museum of Guizhou Education University, Guiyang, Guizhou, China (MGEU, curator Hao Yu).

## Taxonomy

### Family Clubionidae Wagner, 1887

#### Genus Pristidia

Taxon classificationAnimaliaAraneaeClubionidae

Deeleman-Reinhold, 2001

F78D00BB-F5ED-5EE8-BE16-E44947FED4DA

##### Type species.

*Pristidia prima* Deeleman-Reinhold, 2001

##### Diagnosis.

For details see Deeleman-Reinhold (2001) and Yu et al. (2017).

##### Composition and distribution.

*Pristidia longistila* Deeleman-Reinhold, 2001 from Borneo, *P. prima* Deeleman-Reinhold, 2001 from Thailand, Malaysia and Indonesia (Sumatra, Java), *P. secunda* Deeleman-Reinhold, 2001 endemic to Sumatra, *P. viridissima* Deeleman-Reinhold, 2001 widespread from Thailand to Borneo, *P. ramosa* Yu, Sun & Zhang, 2012 and *P. cervicornuta* from China.

#### Pristidia cervicornuta

Taxon classificationAnimaliaAraneaeClubionidae

Yu, Zhang & Chen, 2017

88918EA1-93D2-5EBC-A68A-E467C68EDB30

**Figure 2.**
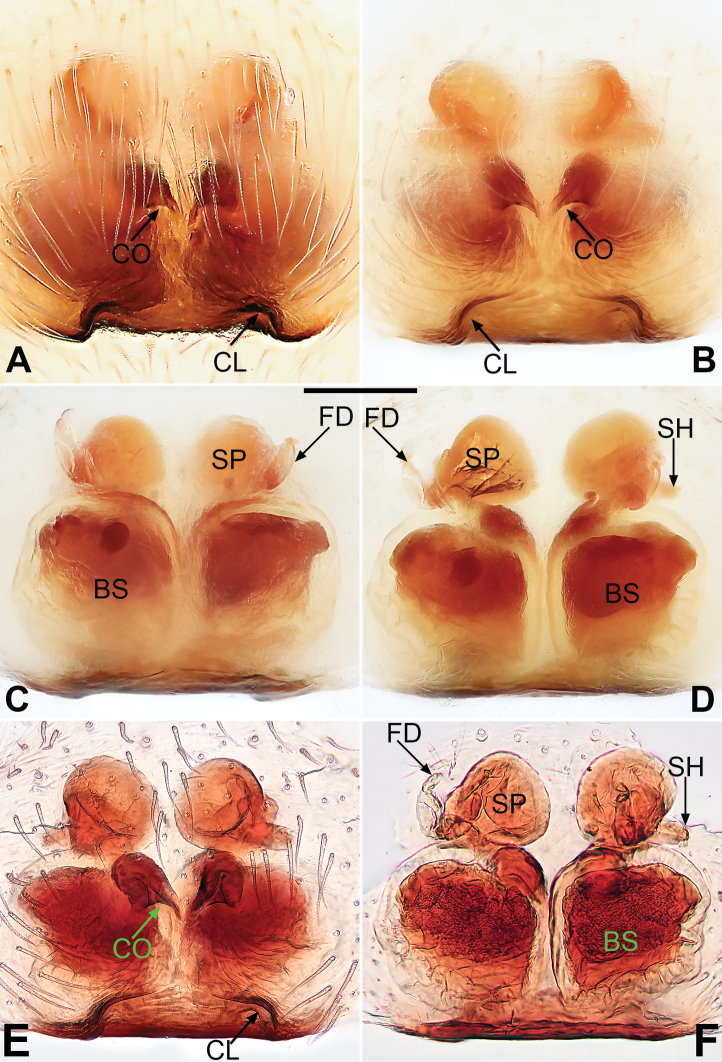
*Pristidia cervicornuta*, female (MGEU-PRI-18-002, **A**) and female (MGEU-PRI-18-001, **B–F**). **A** Epigyne, intact, ventral view **B** Epigyne, cleared, ventral view **C** Vulva, cleared, dorsal view **D** Vulva, cleared, dorsal view **E** Epigyne, cleared, ventral view **F** Vulva, cleared, dorsal view. Scale bars: 0.1 mm. Abbreviations: CL, cleft; CO, copulatory opening; FD, fertilization duct; SH, spermathecal head; SP, spermatheca; BS, bursa.

**Figure 3.**
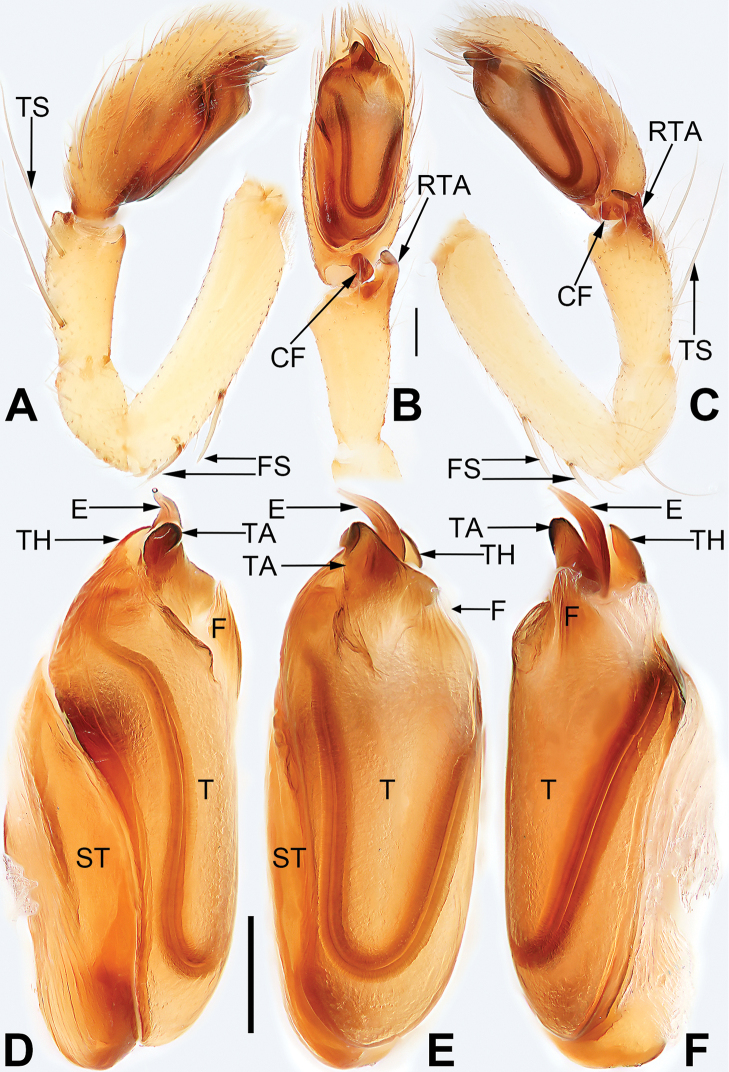
Male left palp of *Pristidia cervicornuta* (MGEU-PRI-18-018). **A** Prolateral view **B** Ventral view **C** Retrolateral view **D** Bulb, prolateral view **E** Same, ventral view **F** Same, retrolateral view. Scale bars: 0.1 mm (equal for **A–C**, equal for **D–F**). Abbreviations: CF, cymbial flange; E, embolus; F, flakelet; FS, femoral spines; RTA, retrolateral tibial apophysis; ST, subtegulum; T, tegulum; TA, tegular apophysis; TH, tegular hump; TS, tibial spines.

**Figure 4.**
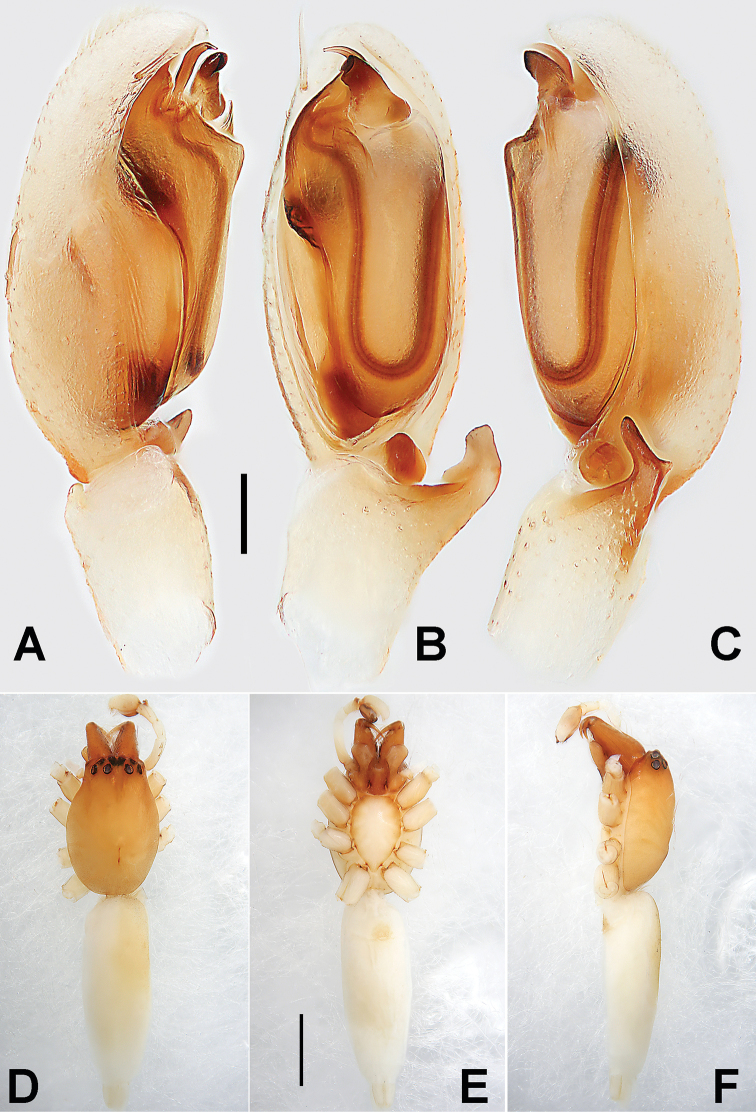
Holotype male of *Pristidia cervicornuta*. **A** Left palp, prolateral view **B** Same, ventral view **C** Same, retrolateral view **D** Male habitus, dorsal view **E** Same, ventral view **F** Same, lateral view. Scale bars: 0.1 mm (equal for **A–C**); 1 mm (equal for **D–F**).

**Figure 5.**
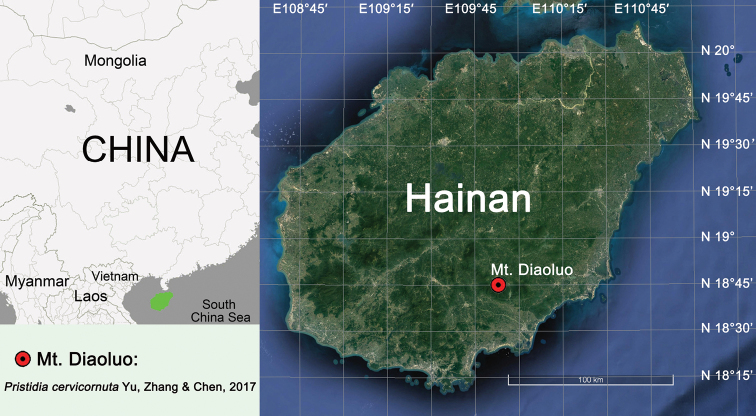
Type locality of *Pristidia cervicornuta* (red circle).

Figs 1 , 2 , 3 , 4 , 5

Pristidia cervicornuta Yu, Zhang & Chen, 2017: 413, f. 18 (♂).

##### Material examined.

CHINA • 17♀(MGEU-PRI-18-001~017) and 15♂ (MGEU-PRI-18-018~032) Hainan Province, Diaoluo Mountains Nature Reserve, Taiping farm; 18°48'15.22"N, 109°52'8.94"E; 380 m; beating of bush, 15 Apr. 2018, Qian Yu leg.; • 2♂ (**Types**); same locality; 18°48'12.16"N, 109°52'5.42"E; 6 Oct. 2009, Hao Yu and Zhenyu Jin leg; beating of shrubs.

##### Diagnosis.

Females of *P. cervicornuta* are similar to those of *P. ramosa* (the other only *Pristidia* species in China: Yu et al. 2012: 45, figs 9–11, 15–16) by the epigynal plate having 2 clefts situated at the posterior margin; they also resemble those of *P. secunda* (Deeleman-Reinhold 2001: 186, figs 191, 192) in the general shape of the vulva, but can be differentiated from *P. ramosa* by lacking atrium (Fig. 2A, B, E) (vs. atrium present in *P. ramosa*), and can be easily distinguished by the indistinct insemination ducts (Fig. 2C, D, F) (vs. long insemination ducts in *P. ramosa* and *P. secunda*), and by the copulatory openings located in the middle of the epigynal plate (Fig. 2A, B, E) (located on posterior margin in *P. ramosa* and *P. secunda*). Males of *P. cervicornuta* can be easily recognized by the distally forked, antler-shaped retrolateral tibial apophysis and by the thick, semitransparent, thumb-shaped tegular apophysis.

##### Description.

***Female*** (MGEU-PRI-18-001) (Fig. 1A–C). Total length 5.25; carapace 1.90 long, 1.29 wide; abdomen 3.21 long, 1.65 wide.

*Carapace* yellow, without distinct pattern. Fovea red. In dorsal view, anterior eye row (AER) slightly recurved, posterior eye row (PER) almost straight, PER wider than AER. Eye sizes and interdistances: anterior median eyes (AME) 0.07, anterior lateral eyes (ALE) 0.05, posterior median eyes (PME) 0.10, posterior lateral eyes (PLE) 0.06; distance between AMEs (AME–AME) 0.02, distance between AME and ALE (AME–ALE) 0.04, distance between PMEs (PME–PME) 0.13, distance between PME and PLE (PME–PLE) 0.04. Length of median ocular quadrangle (MOQ) 0.26, MOQ anterior width 0.20, MOQ posterior width 0.38. *Chelicerae* protruding and robust, with long and red wine-coloured fangs, with 3 teeth on promargin and 2 on retromargin. Labium and endites brown. Sternum 1.06 long, 0.71 wide.

*Abdomen* lanceolate, white, with inconspicuous anterior tufts of sparse hairs, dorsum without pattern; venter white, with several pairs of inconspicuous muscular dots.

*Legs* uniformly light yellow. Leg length: I 5.13 (1.28, 0.71, 1.75, 0.93, 0.46), II 5.11 (1.43, 0.67, 1.69, 0.81, 0.51), III 4.23 (0.97, 0.49, 1.12, 1.21, 0.44), IV 5.85 (1.57, 0.49, 1.51, 1.75, 0.52).

*Epigyne* (Fig. 2B–F). Epigynal plate slightly shorter than wide, margin not rebordered; posterior margin concaved in the middle, forming shallow depression; 2 clefts located at lateral borders of the depression; copulatory openings distinct and heavily sclerotised, located in the middle of the plate. Insemination ducts short and indistinct; spermathecae located anteriorly; spermathecal head small tubercle-like, located on lateral side of spermatheca; bursal surface hyaline, wrinkled and ribbed, inside pigmented and sclerotised; both spermathecae and bursae are subglobular; acicular fertilisation ducts located on the dorso-lateral sides of spermathecae.

***Male*** (MGEU-PRI-18-008) (Fig. 1D–F). Total length 4.06; carapace 1.62 long, 1.28 wide; abdomen 2.25 long, 0.94 wide. Eye sizes and interdistances: AME 0.08, ALE 0.08, PME 0.11, PLE 0.09; AME–AME 0.04, AME–ALE 0.03, PME–PME 0.13, PME–PLE 0.05. MOQL 0.24, MOQA 0.18, MOQP 0.36. Sternum 0.91 long, 0.66 wide. Measurements of legs: I 5.38 (1.26, 0.45, 1.92, 1.16, 0.59), II 5.33 (1.56, 0.45, 1.73, 1.12, 0.47), III 4.39 (1.47, 0.45, 0.76, 1.21, 0.51), IV 6.27 (1.72, 0.54, 1.61, 1.87, 0.54). General characters as in female, but slightly smaller in size and darker in colour.

*Palp* (Fig. 3A–F). See Yu et al. (2017).

##### Comments.

There is almost no difference between the holotype male (Fig. 4A–F) and the newly collected male specimen in the present study. However, two characters of the bulb were not presented in the original description. Additionally, some spines and hairs are lost in holotype male (Fig. 4A–C). Consequently, a fuller description is provided here: the tegular hump (TH) is represented by an enlarged flange, hidden behind the embolus (E) and tegular apophysis (TA); a translucent flakelet (F) located at distal-retrolateral position of tegulum (T) (approximately 1 o’clock of tegulum), the flake is subtriangular with a membranous and blunt apex; the tibia has two long dorsal spines (TS) originating from trisection; the femur bears two short dorsal spines (FS) originating from its proximal part.

##### Natural history.

*Pristidia cervicornuta* inhabits forest located in low elevation areas on Mt. Diaoluo. The male holotype was obtained from shrubs in a rubber-tea artificial community and the new materials were collected by beating twigs and branches of bush in an elm forest.

##### Distribution.

Known only from the type locality, Mt. Diaoluo, Hainan, China (Fig. 5).

## Supplementary Material

XML Treatment for Pristidia

XML Treatment for Pristidia cervicornuta
